# Cross-communication between histone H3 and H4 acetylation and Akt-mTOR signalling in prostate cancer cells

**DOI:** 10.1111/jcmm.12299

**Published:** 2014-04-30

**Authors:** Jasmina Makarević, Nassim Tawanaie, Eva Juengel, Michael Reiter, Jens Mani, Igor Tsaur, Georg Bartsch, Axel Haferkamp, Roman A Blaheta

**Affiliations:** Department of Urology, Johann Wolfgang Goethe-UniversityFrankfurt am Main, Germany

**Keywords:** mTOR, HDAC, cross-communication, prostate cancer cells

## Abstract

Molecular tumour targeting has significantly improved anti-cancer protocols. Still, the addition of molecular targeting to the treatment regime has not led to a curative breakthrough. Combined mammalian target of Rapamycin (mTOR) and histone deacetylase (HDAC) inhibition has been shown not only to enhance anti-tumour potential, but also to prevent resistance development seen under mono-drug therapy. This investigation was designed to evaluate whether cross-communication exists between mTOR signalling and epigenetic events regulated by HDAC. DU-145 prostate cancer cells were treated with insulin-like growth factor (IGF) to activate the Akt-mTOR cascade or with the HDAC-inhibitor valproic acid (VPA) to induce histone H3 and H4 acetylation (aH3, aH4). Subsequently, mTOR, Rictor, Raptor, p70s6k, Akt (all: total and phosphorylated), H3 and H4 (total and acetylated) were analysed by western blotting. Both techniques revealed a link between mTOR and the epigenetic machinery. IGF activated mTOR, Rictor, Raptor, p70s6k and Akt, but also enhanced aH3 and aH4. Inversely, IGFr blockade and knock-down blocked the Akt-mTOR axis, but simultaneously diminished aH3 and aH4. VPA treatment up-regulated histone acetylation, but also activated mTOR-Akt signalling. HDAC1 and 2 knock-down revealed that the interaction with the mTOR system is initiated by histone H3 acetylation. HDAC-mTOR communication, therefore, is apparent whereby tumour-promoting (Akt/mTOR^high^, aH3/aH4^low^) and tumour-suppressing signals (Akt/mTOR^low^, aH3/aH4^high^) are activated in parallel. Combined use of an HDAC- and mTOR inhibitor might then diminish pro-tumour effects triggered by the HDAC- (Akt/mTOR^high^) or mTOR inhibitor (aH3/aH4^low^) alone.

## Introduction

The development of targeted drugs has significantly enriched the armamentarium of anti-tumour agents. As the phosphatidyl-inositol-3-kinase (PI3K)/Akt/mammalian target of rapamycin (mTOR) pathway is aberrantly activated in many tumour types, mTOR suppression is regarded to be an attractive approach for cancer therapy [1]. Two mTOR inhibitors, everolimus and temsirolimus, have already been approved for first and second line treatment in patients with metastatic renal cell carcinoma and are currently under investigation for treating other solid tumours.

Unfortunately, although the strategy of mTOR targeting offers significantly improved response rates, it is rarely curative [2]. Presumably, the tumour adapts to chronic drug use, limiting efficacy [3]. In fact, it has recently been demonstrated that long-term treatment with the mTOR inhibitor everolimus causes drug non-responsiveness, demonstrated by reactivation of the tumour growth and invasion program [4,5]. An innovative option to overcome this obstacle might be the additional application of a histone deacetylase (HDAC) inhibitor. Indeed, elevated HDAC expression, associated with reduced histone acetylation, is a typical feature found in many cancer types and responsible for tumour suppressor gene silencing and malignant transformation. Sharma *et al*. have suggested that the therapeutic integration of a HDAC inhibitor in anti-tumour protocols might distinctly improve the strategy to handle advanced malignancy [6].

The HDAC inhibitor valproic acid (VPA), synergized with everolimus has stopped prostate cancer growth and invasion in pre-clinical studies [7,8]. The VPA-everolimus combination does not induce resistance in tumour cells, even after long-term application, although this phenomenon became evident when each drug was applied alone [9]. This finding has prompted speculation that HDAC inhibition not only acts on histone acetylation, but may also interfere with the mTOR signalling pathway. *Vice versa*, interference with the mTOR signalling pathway may inhibit HDAC, preventing the development of resistance relevant feedback loops, seen when just one pathway is targeted. Employing the DU-145 prostate cancer cell line, a possible HDAC-triggered histone acetylation connection to the Akt-mTOR axis was investigated. The question of whether Akt-mTOR-derived signals contribute to modifications of histone acetylation was also investigated.

## Materials and methods

### Cell culture

The human prostate tumour cell line DU-145 was obtained from DSMZ (Braunschweig, Germany). Tumour cells were grown in RPMI 1640 (Gibco/Invitrogen, Karlsruhe, Germany), 10% foetal calf serum, 2% HEPES buffer (1 M, pH 7.4), 2% glutamine and 1% penicillin/streptomycin at 37°C in a humidified, 5% CO_2_ incubator.

### Activation of cell signalling

To activate the Akt-mTOR signalling pathway, DU-145 tumour cells were kept in serum-free cell culture medium overnight and subsequently stimulated for 30 min. with epidermal growth factor (EGF, 100 ng/ml), insulin-like growth factor (IGF, 100 ng/ml) or both EGF and IGF and then subjected to western blotting. Tumour cell lysates were applied to a 7% polyacrylamide gel and electrophoresed for 90 min. at 100 V. The protein was then transferred to nitrocellulose membranes. After blocking with non-fat dry milk for 1 hr, the membranes were incubated overnight using the following monoclonal antibodies: Anti phospho-IGFr directed to the IGF1 receptor, IGF1R (pIGFr; IgG1, Thr 950, clone J95-626; BD Pharmingen, Heidelberg, Germany), Anti mTOR (IgG, clone 7C10), Anti phospho mTOR (pmTOR; IgG, Ser2448, clone D9C2), Anti Rictor (IgG, clone 53A2), Anti phospho Rictor (pRictor; IgG, Thr1135, D30A3), Anti Raptor (IgG, clone 24C12), Anti phospho Raptor (pRaptor; IgG, Ser792), Anti p70s6k (IgG, clone 49D7), Anti phospho p70s6k (pp70s6k; IgG, Thr389, clone 108D2; all: New England Biolabs, Frankfurt, Germany), Anti Akt (IgG1, clone 55), Anti phospho Akt (pAkt; IgG1, Ser472/Ser473, clone 104A282; both: BD Pharmingen), Anti histone H3 (IgG, clone 3H1), Anti acetylated H3 (aH3; IgG, Lys9, clone C5B11), Anti histone H4 (IgG, clone L64C1,), Anti acetylated H4 (aH4; Lys8, polyclonal, IgG; all: New England Biolabs). HRP-conjugated goat antimouse IgG (Upstate Biotechnology, Lake Placid, NY, USA; dilution 1:5000) served as the secondary antibody. The membranes were briefly incubated with ECL detection reagent (ECLTM, Amersham/GE Healthcare, München, Germany) to visualize proteins and then analysed by the Fusion FX7 system (Peqlab, Erlangen, Germany). β-actin (1:1000; Sigma-Aldrich, Taufenkirchen, Germany) served as internal control.

### Histone acetylation

DU-145 tumour cells were kept in serum-free cell culture medium overnight and subsequently stimulated for 24 hrs with the HDAC inhibitor VPA (1 mmol/ml). Western blotting was then carried out using the same antibodies as indicated above.

### IGFr expression

IGFr expression and activity, which is a necessary prerequisite to transduce growth factor-related signalling to the Akt-mTOR pathway was explored by flow cytometry. DU-145 cells were kept in serum-free cell culture medium overnight and then treated with different concentrations of IGF for different time periods. Subsequently, cells were washed in blocking solution (PBS, 0.5% bovine serum albumin) and then incubated for 60 min. at 4°C with phycoerythrin (PE)-conjugated monoclonal antibodies directed against IGFr (IGFr-1; IgG1, clone 1H7; BD Pharmingen) or phospho-IGFr (pIGFr-1, IgG1, Alexa-labelled, clone K74-218; BD Pharmingen). Receptor surface expression was then measured using a FACScan [Becton Dickinson, Heidelberg, Germany; FL-2H (log) channel histogram analysis; 1 × 10^4^ cells/scan]. A mouse IgG1-PE or IgG1-Alexa (MOPC-21; BD Pharmingen) was used as an isotype control. Fluorescence data were related to fluorescence values of the unstimulated controls which were set to 100%.

### IGFr blockade and knock-down studies

To verify that IGFr-triggered Akt-mTOR activation directly contributes to histone modification, IGFr blocking studies were carried out. DU-145 cells were pre-incubated for 60 min. with a function blocking anti-IGFr monoclonal antibody (20 μg/ml, clone 33255.111; Calbiochem-Merck, Darmstadt, Germany) under serum-free conditions. Controls remained untreated. IGF (100 ng/ml) was then added and the following proteins analysed by western blotting: pIGFr, pRaptor, pRictor, pAkt, aH3 and aH4. In addition, IGFr protein synthesis was down-regulated by small interfering RNA (siRNA) directed against IGFr (gene ID: 3480, target sequence: TCGAAGAATCGCATCATCA; Qiagen, Hilden, Germany) with a siRNA/transfection reagent (HiPerFect Transfection Reagent; Qiagen) ratio of 1:6. Non-treated cells and cells treated with 5 nM control siRNA (All stars negative control siRNA; Qiagen) served as controls. Subsequently, the consequences of IGFr knock-down for histone H3 and H4 acetylation were analysed by the western blot assay using aH3 and aH4 antibodies, as indicated above.

To explore whether histone acetylation specifically acts on the Akt-mTOR pathway, DU-145 cells were transfected with siRNA directed against HDAC1 (gene ID: 3065, target sequence: CACCCGGAGGAAAGTCTGTTA; Qiagen) or HDAC2 (gene ID: 3066, target sequence: TCCCAATGAGTTGCCATATAA; Qiagen), with a siRNA/transfection reagent (HiPerFect Transfection Reagent; Qiagen) ratio of 1:6. Non-treated cells and cells treated with 5 nM control siRNA (All stars negative control siRNA; Qiagen) served as the controls. Thereafter, aH3, aH4, pIGFr, pRictor, pRaptor and pAkt were analysed by western blotting.

### Statistics

All experiments were performed 3–6 times. Statistical significance of the flow cytometry experiments was determined with the Wilcoxon–Mann–Whitney *U*-test. Differences were considered statistically significant at a *P* < 0.05.

## Results

### IGFr expression and activity

**Fig. 1:**
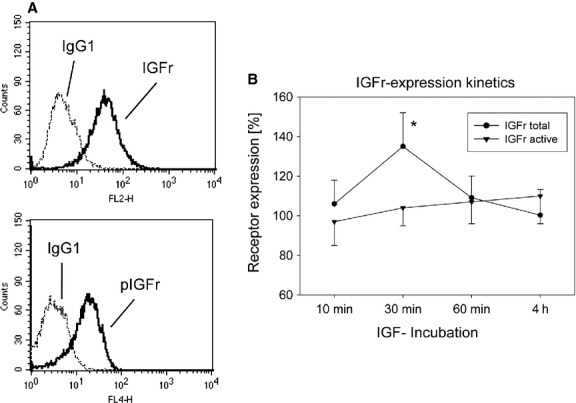
(**A**) FACS analysis of IGFr and pIGFr expression on PC3^par^ *versus* PC3^res^ cells. Cells were washed in blocking solution and then stained with specific monoclonal antibodies as listed in Materials and Methods. To evaluate background staining, goat antimouse IgG1-PE or IgG1-Alexa was used (dotted lines). Fluorescence was analysed using a FACScan flow cytometer. One of three independent experiments. (**B**) Time dependent IGFr (IGFr total) and pIGFr (IGFr active) expression on DU-145 cells. Tumour cells were kept in serum-free cell culture medium overnight and then exposed to 100 ng/ml IGF for different time periods. Thereafter, receptor expression was evaluated by a FACScan flow cytometer, whereby untreated controls were set to 100%. One representative of six experiments is shown. * indicates significant difference to controls.

IGFr surface expression is a necessary prerequisite to growth factor-evoked activation of the Akt-mTOR signalling pathway. Therefore, the content of total membranous IGFr as well as phosphorylated IGFr in the DU-145 cells was evaluated. Figure 1A demonstrates that both receptor types are detectable by flow cytometry. Exposing the cells to 100 ng/ml IGF led to a time-dependent increase in pIGFr with a maximum response after 30 min. (Fig. 1B). Application of 50 ng/ml IGF revealed the same effect, and higher IGF concentrations (200 and 500 ng/ml) did not further increase receptor phosphorylation (data not shown). Therefore, 100 ng/ml IGF was used in subsequent studies and cell signalling was evaluated 30 min. after IGF stimulation.

### mTOR signalling affects histone acetylation

**Fig. 2:**
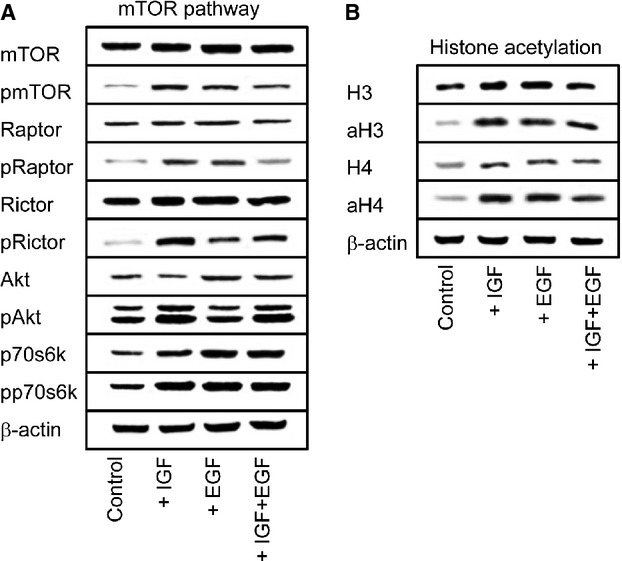
Growth factor induced alterations of proteins related to the mammalian target of rapamycin (mTOR) pathway (**A**) and to epigenetic processes (**B**). DU-145 cells were kept in serum-free cell culture medium overnight and subsequently stimulated for 30 min. with epidermal growth factor (EGF, 100 ng/ml), insulin-like growth factor (IGF, 100 ng/ml) or both EGF and IGF. Controls remained unstimulated. Cell lysates were then subjected to SDS-PAGE and blotted on the membrane incubated with the respective monoclonal antibodies listed in methods. β-actin served as the internal control. The figure shows one representative from three separate experiments.

Activation of DU-145 cells with IGF caused a distinct increase in pmTOR, pRaptor, pRictor, pAkt and pp70s6k, compared to the controls (Fig. 2A). IGF also elevated histone H3 and H4 acetylation (Fig. 2B). Further experiments demonstrated that mTOR-histone linkage is not an IGF specific phenomenon, as the growth factor EGF triggered similar effects, except for pAkt, which was not modified. No additive effects were seen in the presence of both EGF and IGF (Fig. 2B). pRaptor was even diminished, compared to cells treated with IGF or EGF alone. It has recently been shown that activating the cells by IGF may suppress signalling molecules of the EGFr pathway [10]. The reciprocal crosstalk may, therefore, create a situation whereby simultaneous targeting of both IGFr and EGFr neutralizes effects seen in the presence of just one growth factor.

**Fig. 3:**
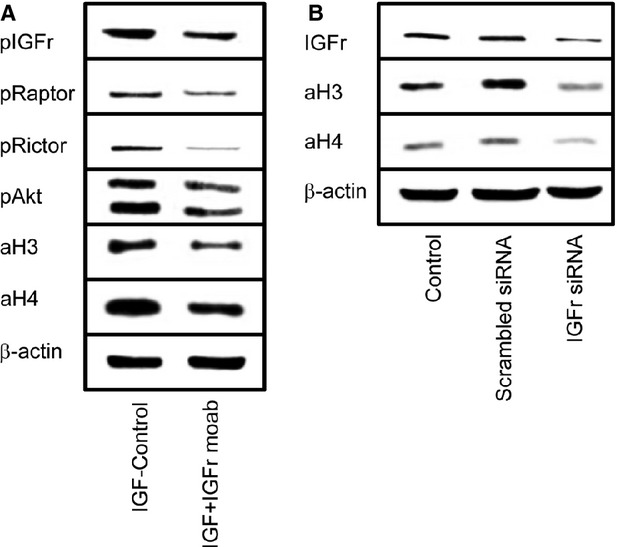
IGFr blocking studies. (**A**) DU-145 cells were pre-incubated for 60 min. with a function blocking anti-IGFr monoclonal antibody and then treated with IGF (100 ng/ml). Controls remained untreated. Cells were then subjected to western blot analysis and histone H3 and H4 acetylation and activation of mammalian target of rapamycin (mTOR) relevant signalling proteins were explored. (**B**) IGFr protein synthesis was down-regulated by small interfering RNA directed against IGFr. Non-treated cells and cells treated with scrambled siRNA served as controls. Histone H3 and H4 acetylation was then analysed by western blotting. One representative from three separate experiments.

To investigate whether H3 and H4 acetylation is a down-stream event following IGFr-Akt-mTOR activation, tumour cells were incubated with a function blocking antibody directed against IGFr. Receptor blockade not only diminished pIGFr, pRaptor, pRictor and pAkt, but also lowered histone H3 and H4 acetylation (Fig. 3A). To gain more detail, DU-145 cells were treated with IGFr siRNA to reduce the IGFr protein content. Similar to the blocking experiments, the strategy resulted in the down-regulation of H3 and H4 acetylation, which was most prominent with respect to aH3 (Fig. 3B).

### Histone acetylation affects mTOR signalling

**Fig. 4:**
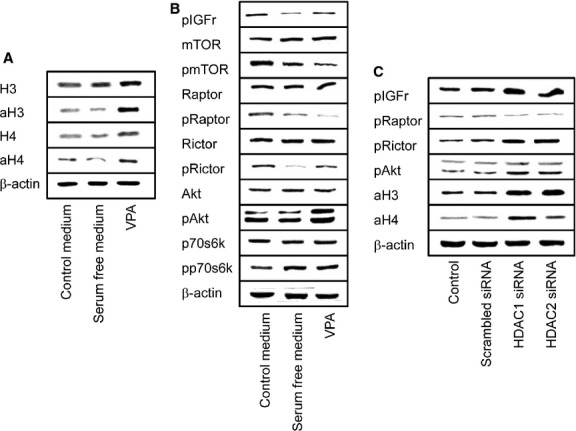
Valproic acid (VPA) induced alterations of histones H3 and H4 (**A**) and of proteins related to the mammalian target of rapamycin (mTOR) pathway (**B**). DU-145 cells were kept in serum-free cell culture medium overnight and subsequently treated with VPA (1 mmol/ml). Controls did not receive VPA. A second control consisted of DU-145 cells cultured in serum containing medium. Cell lysates were then subjected to SDS-PAGE and blotted on the membrane incubated with the respective monoclonal antibodies listed in the methods part. β-actin served as the internal control. (**C**) siRNA knock-down. HDAC was down-regulated by small interfering RNA directed against HDAC1 or HDAC2. Non-treated cells and cells treated with scrambled siRNA served as the controls. Histone H3 and H4 acetylation and alterations of the mTOR pathway were then analysed by western blotting. The figure shows one representative from three separate experiments.

To investigate whether mTOR-histone linkage is of a uni- or bidirectional nature, the tumour cells were then treated with VPA instead of IGF. Experiments were carried out under serum-free conditions to avoid undesired side effects from serum-derived growth factors. As expected, VPA enhanced the expression level of aH3 and aH4 (Fig. 4A). However, it also altered signalling behaviour of the mTOR axis. pmTOR and pRaptor were reduced, whereas pIGFr, pRictor and pAkt were all enhanced by VPA. No differences to controls were seen on pp70s6k (Fig. 4B).

Finally, the mTOR pathway was investigated to determine whether it can specifically be modified *via* HDAC. HDAC1 or HDAC2 was knocked down by siRNA and the consequences for mTOR activation analysed. HDAC1 knock-down correlated with a distinct up-regulation of aH3 and aH4, whereas HDAC2 knock-down predominantly elevated aH3 (Fig. 4C). Both HDAC1 and HDAC2 knock-down led to an up-regulation of pAkt, pRictor and pIGFr and loss of pRaptor (Fig. 4C).

## Discussion

Activating the mTOR pathway by IGF induced distinct alterations of the histone acetylation level in DU-145 tumour cells. The link was specific, as demonstrated by the IGFr blocking and knock-down studies. mTOR activation correlated with aH3 and aH4 expression in a positive manner. Enhanced histone acetylation was found in IGF-stimulated cells, whereas mTOR pathway suppression evoked a distinct acetylation loss. This relationship is important from a clinical viewpoint. Although mTOR inhibitors are routinely used to treat patients suffering from renal cancer, substantial anti-tumour response has only been experienced by a subset of patients [11]. Limitations of everolimus or temsirolimus were also seen in patients with other solid tumours [12,13], making it likely that the limited efficacy of an mTOR-based regimen is a general problem.

The reason for mTOR inhibitors not living up to expected potential has not been fully elucidated. However, it has been suggested that chronic drug treatment creates undesired feedback loops, reactivating the Akt-mTOR pathway and enabling tumour cells to restart their growth program [14,5]. Apart from the concept of acquired resistance under long-term drug treatment, the existence of an mTOR-histone network, as shown in the present investigation, points to a further mechanism, possibly responsible for the limited potential of mTOR inhibitors. Indeed, mTOR blockade very rapidly induced the removal of acetyl groups on histones H3 and H4. A similar phenomenon has recently been observed in rhabdoid tumours [15], indicating that such cross-linking is not restricted to prostate cancer.

Histones are critically involved in cancerogenesis and cancer recurrence [16,17], whereby deacetylation of the histones H3 and H4 has been associated with cell proliferation, motility, invasion and clonal expansion [18,19]. Hence, diminishing the aH3 and aH4 level may counteract the positive effects of mTOR inhibitors exerted by deactivating the Akt-mTOR cascade. This hypothesis is quite different from the acquired resistance paradigm. In fact, the immediate action on epigenetic molecules following mTOR suppression suggests that the anti-tumour potential of mTOR inhibitors is restricted *per se*, regardless of drug exposure time.

Manipulating the DU-145 cells by blocking HDAC and increasing histone acetylation also established communication with Akt-mTOR. Similar to the IGF stimulation assay, a positive correlation was recorded between aH3/aH4 expression and phosphorylation of Akt, Rictor and IGFr. However, a negative association was seen between aH3/aH4 expression and pmTOR and pRaptor, in contrast to the results achieved *via* IGF activation. mTOR is composed of two complexes, mTORC1 with Raptor as the prominent protein and mTORC2, which predominantly contains Rictor. As the inverse relationship became evident with respect to Raptor but not to Rictor, the mTOR data are most probably attributed to the Raptor molecule. We, therefore, assume that a bidirectional interaction exists between the mTOR system and the epigenetic machinery in cancer cells, whereby the divergent Raptor behaviour points to differences in how both systems communicate. Adjustment of intracellular metabolic pathways is thought to be regulated by interplay between IGFr, Akt-mTOR and de/acetylation events, which supports this assumption [20]. Further experiments are intended to obtain more detailed insight into the fine tuning of the IGFr-triggered TOR histone compared to the HDAC-triggered histone-mTOR connection.

Valproic acid serves as a pan HDAC inhibitor. To allow a more precise analysis of the mode of action underlying HDAC-triggered mTOR modulation, HDAC1 or HDAC2 was knocked down. Both strategies modified the mTOR pathway. However, H4 acetylation was elevated only in those cells incubated with HDAC1 siRNA, whereas aH3 was up-regulated by both HDAC1 and HDAC2 siRNA. This observation may point to a major role of aH3 in initiating the link to mTOR. In good accordance, the HDAC inhibitor vorinostat has been shown to modify IGFr and Akt phosphorylation in endometrial cancer cells by acetylating histone H3 [21].

Valproic acid evoked a scenario similar to the one observed with IGF or IGFr-Akt-mTOR blockade. Anti-tumour effects, reflected by histone acetylation, were paralleled by pro-tumour mechanisms, *i.e*. activation of Akt, Rictor and IGFr. The pro-tumour action is critical and may diminish the therapeutic potential of VPA. Indeed, an *in vivo* renal cell carcinoma study showed that 50% of animals treated with VPA did not respond because Akt was almost completely phosphorylated during therapy [22]. In a similar fashion, VPA has led to hyperacetylation of histone H3 and subsequent Akt activation in cervical cancer and neuroblastoma cells [23,24], making it plausible that activating the Akt pathway under VPA might be common for several tumour types. The risk of diminished anti-tumour activity should therefore be kept in mind whenever initiating clinical trials with HDAC blocking compounds [25], particularly as Akt phosphorylation is inducible by different HDAC inhibitors with variable chemical structures [24].

When discussing the role of VPA, it should be noted that VPA has been shown to deactivate Akt as well as p70s6k in another study [8], contradicting the present finding. However, VPA was applied for 24 hrs in the present experimental setting, whereas long-term application for 3 and 5 days was carried out in the other study. This is important, as VPA's effects may, at least partially, depend on drug exposure time [26]. In addition, and quite relevantly, the DU-145 tumour cells were treated with VPA under serum-free conditions to prevent unspecific activation of the molecular machinery. Therefore, the present model may more precisely reflect VPA's mode of action.

Valproic acid acted on aH3, aH4 and pRaptor in a positive (tumour-suppressive) fashion, on pIGFr, pRictor and pAkt in a negative (tumour-promoting) fashion, whereas blocking the IGF-driven mTOR pathway induced tumour-suppressive effects on pIGFr, pRictor, and pAkt, but induced tumour-promoting action on aH3 and aH4. Therefore, the mTOR/HDAC inhibitor combination might create a molecular scenario, where particular pro-tumour effects exerted by one drug will be negated by a partner drug. This means loss of aH3 and aH4 *via* mTOR blockade could be opposed by VPA, and elevated Akt phosphorylation *via* VPA could be counter-regulated by mTOR inhibition. In good accordance with this hypothesis, the combination of the HDAC inhibitor panobinostat with everolimus has diminished tumour cell proliferation *in vitro* and *in vivo* to a greater extent than either drug alone [27]. Combining panobinostat with the dual PI3K-mTOR inhibitor, BEZ235, also increased anti-tumour activity compared to separate application [28]. HDAC- plus mTOR inhibition was also highly effective in stopping the metastatic activity of cancer cells [7]. Finally, acquisition of resistance under VPA or everolimus is prevented when exposing the tumour cells to both drugs concurrently [9]. Applying this mechanism, a dual-acting compound has been constructed which disrupts cancer networks *via* potent inhibition of the Akt-mTOR pathway and epigenetic effects of HDAC [29].

Overall, evidence has been presented that HDAC-mTOR cross-linking exists in tumour cells, and combined application of an HDAC- and mTOR inhibitor might overcome limitations encountered with a mono-drug regime. This conclusion is drawn from *in vitro* studies. To evaluate the therapeutic relevance, an animal model would be required. Promising results have come from a phase I study in patients with relapsed or refractory Hodgkin and non-Hodgkin lymphoma where panobinostat and everolimus in combination showed favourable clinical effects [30]. It might be worthwhile to transfer this treatment strategy to patients suffering from solid tumours. Treatment regimes may not necessarily be restricted to incorporating the substances investigated here. Rather, alternate compounds targeting the same pathways should also be considered to discover combinations with highest tolerability and lowest toxicity.
